# Characterization of the central nervous system penetrant and selective purine P2X7 receptor antagonist JNJ-54175446 in patients with major depressive disorder

**DOI:** 10.1038/s41398-023-02557-5

**Published:** 2023-07-24

**Authors:** Kasper Recourt, Peter de Boer, Peter van der Ark, Heike Benes, Joop M. A. van Gerven, Marc Ceusters, Luc van Nueten, Wayne C. Drevets, Anindya Bhatacharya, Michael Browning, Gabriel E. Jacobs

**Affiliations:** 1 Centre for Human Drug Research, Leiden, the Netherlands; 2 Department of Psychiatry, Leiden University Medical Center, Leiden, the Netherlands; 3 Janssen Research and Development, a Division of Janssen Pharmaceutica N.V, Beerse, Belgium; 4 Janssen Research and Development LLC, La Jolla, CA USA; 5 University of Oxford, Oxford, UK; 6 Oxford Health NHS Trust, Oxford, UK

**Keywords:** Depression, Clinical pharmacology

## Abstract

JNJ-54175446 is a selective purine P2X7 receptor (P2X7R) antagonist that attenuates microglial IL-1β/IL-18 release. In healthy volunteers, JNJ-54175446 suppressed peripheral interleukin (IL)-1β release, and attenuated dexamphetamine-induced improvements of mood and (visuo)motor performance in a human dexamphetamine-challenge paradigm. In depression, P2X7R inhibition may dampen immune-related dysregulation of mood. These results suggest that the impact of P2X7R inhibition is most prominent in situations where mood regulation is disrupted. Total sleep deprivation (TSD) results in an acute emotional perturbation, which yields a transient antidepressant effect. In the current study, TSD was applied as a behavioral challenge to investigate whether such effects could be modulated by JNJ-54175446. This was a double-blind, placebo-controlled, randomized study to assess the safety and pharmacokinetics of JNJ-54175446 and explore its effects in patients with single episode and recurrent major depressive disorder (MDD) (*N* = 69) and baseline total Inventory of Depressive Symptomatology Clinician Rated (IDS-C) > 30. Patients were randomized to receive JNJ-54175446 throughout the 10-day treatment period, placebo for days 1–3 followed by JNJ-54175446 or placebo throughout. All patients underwent 36 h of TSD starting on day three until the evening of day four. The early start group was hypothesized to experience a reduced effect from TSD whilst the late starting group was hypothesized to experience prolonged effects from the TSD. JNJ-54175446 was well-tolerated and adverse events were mild to moderate. JNJ-54175446 reduced IL-1β release by LPS-stimulated peripheral white blood cells in the presence of the P2X receptor agonist benzyl adenosine triphosphate (BzATP). JNJ-54175446 did not have a significant effect on mood as assessed using the Hamilton Depression Rating Scale, 17 items (HDRS17) and the Self-rated Quick Inventory of Depressive Symptoms (QIDS-SR). However, JNJ-54175446 blunted an acute reduction of anhedonia that occurred as a result of TSD, assessed by the Snaith-Hamilton Pleasure Scale (SHAPS) and the Probabilistic Instrumental Learning Task (PILT).

## Introduction

The purine P2X7 receptor (P2X7R) is one of seven identified subtypes of adenosine triphosphate (ATP)-gated P2X ion channels present on various human cell types. The P2X7R is predominantly expressed in central nervous system (CNS) in microglial cells and peripherally in monocytes [1]. Since ATP has a relatively low affinity for the P2X7R, it is considered a ‘silent receptor’ under physiological, non-stressed cellular conditions [2]. In contrast, when potentially noxious cellular stimuli such as oxidative stress, inflammation, or cellular injury [3] occur, sufficient ATP is released to activate P2X7Rs. Subsequent inflammasome activation is initiated, and as a result, pro-inflammatory cytokines including interleukin (IL)-1β are released in both the CNS and periphery [4–10]. In the CNS, this P2X7–IL-1β pathway has been demonstrated to play a key role in neuroinflammation [2, 11–15].

Several lines of evidence implicate neuroimmune mechanisms in the pathophysiology of mood disorders [16]. In patients with both recurrent and persistent mood disorders, alterations in the innate immune system and inflammatory responses, including increased concentrations of circulating cytokines such as IL-1β, have been observed [17–19]. Also, the comorbidity of MDD with chronic systemic inflammatory disease further supports a pathophysiological role for inflammation in mood disorders [20, 21]. Conversely, antidepressant drugs have been demonstrated to decrease peripheral cytokine levels in some, but not all studies in MDD patients [22–24] and adjunctive treatment with immunomodulatory agents such as NSAIDs or cytokine inhibitors has produced mood improvement in some but not all studies of depression [25, 26].

Because of the well-documented role of the P2X7R in IL-1β release, it is hypothesized that P2X7R antagonists may be beneficial for depression associated with neuroinflammation. Iwata and colleagues were the first to show that chronic stress in rodents results in the activation the P2X7R and NLRP3 inflammasome-related release of IL-1β in hippocampus [27]. Furthermore, antagonism of central but not peripheral P2X7Rs was found to reverse stress-induced anhedonia in rodents [28, 29]. A possible mechanism of P2X7R-mediated local effects derives from studies in pain where (tetanic) neuronal stimulation results in the output of glutamate and ATP from nerve endings, activating astroglia while recruiting microglia, which locally release IL-1β that stimulates synaptic strength by the recruitment of metabotropic glutamate receptor subtype 1 receptors [30]. Together these data support the hypothesis that P2X7R may afford a novel therapeutic target in the treatment of mood disorders in general and particularly in recurrent forms associated with chronic stress.

Currently, a number of CNS-penetrant high affinity and selective P2X7R antagonists, such as JNJ-54175446 and JNJ-55308942, are in development for the treatment of unipolar and bipolar mood disorders [31–33]. Preclinical experiments with these compounds demonstrated a reduction of ex-vivo lipopolysaccharide (LPS)-induced IL‐1β release in human monocytes and rat microglia, functional blockade of benzyl (Bz)‐ATP-induced IL‐1β release in rodent brains and reversal of dexamphetamine-induced hyperlocomotion in rodents [2, 34]. In addition, P2X7 knockout mice demonstrated decreased dexamphetamine-induced hyperlocomotion and IL‐1β release in the brain [35, 36], and reduced depressive-like behaviors in the automated tail suspension test, forced swim test and post-stress sucrose preference test, compared with wild type mice [37, 38].

In healthy human studies, JNJ-54175446 demonstrated blood-brain barrier penetration and a clear dose-receptor occupancy relationship in a range of 5–300 mg using positron emission tomography, and was safe and well tolerated [39]. Furthermore and consistent with preclinical experiments, JNJ-54175446 blunted peripheral IL-1β increase in an ex-vivo LPS challenge paradigm at doses ranging from 50 to 600 mg [39] and attenuated dexamphetamine-induced improvements of (visuo)motor performance and sustained attention, while it altered subjective mood elevating effects of dexamphetamine in healthy volunteers [40]. Although JNJ-54175446’s mood modulating effects in animals and healthy humans seem encouraging considering its anticipated clinical indication area, experimental animal models are notorious for their lack of predictive validity for drugs with non-monoaminergic modes of action, and pharmacological challenges in healthy humans are limited by their inability to model human mood disorders in all their complexity. Therefore, the potential impact of JNJ-54175446 on mood regulation in a relevant target population remains to be explored.

Since the P2X7R is activated under conditions of elevated neuronal activity and/or pathology, pharmacological antagonism is only expected to elicit an effect when the channels are activated by sufficiently high ATP concentrations [3]. Hence, translating JNJ-54175446’s mood modulating effects observed in animal models of depression to a human paradigm is challenging. For this reason, different pharmacological and behavioral challenge models have been employed in previous clinical trials to evaluate the activity of JNJ-54175446 by stimulating human blood samples with lipopolysaccharide (LPS) and 3′-O-(4-benzoylbenzoyl)-ATP (BzATP) [39, 41] or by administering dexamphetamine to healthy volunteers [40]. However, in patients with mood disorders the dexamphetamine challenge raises ethical concerns since it could potentially interact with prescribed antidepressants and lead to unwanted adverse events. To allow for concurrent demonstration of mood modulation and changes in cytokine release by JNJ-54175446 following P2X7 stimulation, total sleep deprivation (TSD) can be applied as a behavioral challenge model and a proxy for noxious cellular stimuli that are relevant for mood regulation.

TSD and sleep restriction have been shown to increase ATP levels resulting in an increase of peripheral cytokine levels including IL-1β [42–44]. Furthermore, TSD is followed by a rapid, but transient recovery of depressive symptoms in 40–60% of patients with MDD. This effect generally resolves after a single post-TSD night of recovery sleep, with on average 83% of patients relapsing [45–49]. After this night of recovery sleep, the increased cytokine levels also return to baseline [44], which is accompanied by a significant improvement of both subjective and objective mood-related parameters, even in patients considered treatment resistant before the TSD. Combining TSD with antidepressants or lithium can sustain the improvement of the mood state for weeks to months [50–55]. The mechanism underlying the transient antidepressant effect of TSD remains unknown, but it is hypothesized that sleep deprivation acutely hyperactivates the inflammatory mechanisms, which in some MDD patients appear to be under chronic low-level tonic stimulation [56]. Based on this we conclude that TSD acutely increases cytokine levels [57] which, in turn ameliorates depressive symptoms [58]. This led us to hypothesize that the effect of TSD on MDD symptoms may be modulated by blunting the acute increase of cytokine levels with P2X7 antagonists, making it a suitable clinical behavioral challenge model to assess the efficacy of JNJ-54175446.

Although data from both preclinical and healthy volunteer studies with IL-1β as peripheral pharmacological biomarker for P2X7R antagonism seem encouraging, JNJ-54175446’s mood modulating effects are yet to be established in a target patient population such as MDD. The use of TSD to produce a transient change in mood in patients with MDD conceivably may serve as a probe of the effect of a physiological stressor on mood. In this context JNJ-54175446 is expected to modulate and potentially reduce the effect of TSD on mood and mood-related phenomena. It was hypothesized that TSD-associated acute mood enhancement would be attenuated in MDD patients who had received JNJ-54175446 during the 3 days prior to the TSD challenge, as the acute effects of elevated cytokine levels would be blunted by JNJ-54175446. Conversely, if patients only received JNJ-54175446 *after* TSD, it was hypothesized that the return of a tonic neuroinflammatory state after recovery sleep could potentially be prevented by P2X7R-blockade, which in turn could lead to prolongation of the acute TSD-associated mood enhancement.

The main objective of the current study was to investigate the safety and pharmacokinetics of JNJ-54175446 in MDD, as well as to perform a proof-of-concept exploration of its potential effects on mood-related phenomena by applying TSD in a relevant clinical population.

## Material and methods

### Study design

This was a randomized, double-blind, placebo-controlled, multicentre, safety and tolerability study that took place in the Netherlands and Germany. The study involved TSD as an exploratory proof-of-concept intervention to induce acute mood improvement, which was preceded and/or followed by JNJ-54175446 and/or placebo. The study was performed in accordance with the Declaration of Helsinki and Good Clinical Practice (GCP). Ethical and regulatory approval was obtained in each participating country by the Stichting Beoordeling Ethiek Biomedisch Onderzoek Assen and the Ethikkommission an der medizinischen Fakultät der Universität Rostock. The study was registered on Clintrials.gov under NCT02902601. Informed consent was obtained from all patients before any study activities took place. After the inclusion and exclusion criteria had been confirmed, patients were randomly assigned to receive either JNJ-54175446 throughout the 10-day treatment period (Group A; early start group), placebo for days 1–3 followed by JNJ-54175446 on days 4–10 (Group B: late start group) or placebo throughout the 10 day period (Group C placebo group) in a 3:3:2 ratio. Patients and investigator/site staff were blinded to treatment assignment and randomization was stratified for patients who were treatment naive or treated with SSRI monotherapy. The design is clarified in Table 1.

**Table 1:** Treatment groups (group A JNJ-54175446, group B JNJ-54175446 post TSD, group C placebo).

**Group**	**Day 1**	**Day 2**	**Day 3**	TSD	**Day 4**	Recovery sleep	**Day 5–10**
A (*N* = 24)	JNJ 600 mg	JNJ 150 mg	JNJ 150 mg		JNJ 150 mg		JNJ 150 mg
B (*N* = 24)	Placebo	Placebo	Placebo		JNJ 600 mg		JNJ 150 mg
C (*N* = 16)	Placebo	Placebo	Placebo		Placebo		Placebo

*JNJ* JNJ-54175446, *TSD* total sleep deprivation.

### Population

Male and female patients aged 18–64 years with a body mass index (BMI) between 18 and 32 kg/m^2^ who met the DSM-IV or -5 diagnostic criteria for single episode or recurrent MDD without psychotic features were included. The diagnosis was confirmed by the MINI 6.0 and documentation on the medical history was obtained from their attending psychiatrist, primary care physician or psychotherapist. The Clinician Rated Inventory of Depressive Symptomatology (IDS-C30) total score was required to be ≥30 at screening. Patients were either medication-free or treated with at most one SSRI during a minimum of 6 weeks and a maximum of 6 months. Sertraline was not allowed as concomitant medication, as in vitro data suggested JNJ-54175446 may induce CYP2D6, for which sertraline is a substrate. Patients were assessed to be medically stable on the basis of clinical laboratory tests, medical history, vital signs, and 12-lead ECG performed at screening and baseline. Patients with a current or previous diagnosis of a psychotic disorder, bipolar disorder, substance use disorder, an eating disorder, mental retardation, cluster B personality disorder, suicidal ideation with intent to act within the past 6 months, a history of suicidal behavior within the past year, or a concurrent sleeping disorder were excluded. Patients who had failed more than two antidepressant drugs with different modes of action during the current or in a previous depressive episode, were excluded. Patients were tested for drug abuse and pregnancy during screening and at the start of the drug administration period.

### Dose selection

In an earlier single ascending dose study with JNJ-54175446 in healthy male volunteers, full antagonism of peripheral ex-vivo IL-1β stimulation was demonstrated following single doses of ≥50 mg JNJ-54175446 [39]. A follow-up multiple ascending dose study investigated the cognitive, psychomotor, neurophysiological and subjective drug effects using a CNS test battery. JNJ-54175446 attenuated dexamphetamine-induced motor improvements for finger tapping and adaptive tracking at 300 mg and 450 mg, and at 300 mg but not at 450 mg, respectively [40]. Combined, this served as basis for selecting a dose of 150 mg q.d. for the current study, following a single loading dose of 600 mg on day 1 and 150 mg q.d. from day 2, which was predicted to result in a mean plasma concentration above the EC90 within the first day and reach steady state between day 2 and 3. Therefore, group A received a loading dose of 600 mg JNJ-54175446 on day 1, followed by 150 mg JNJ-54175446 once daily through day 10. Group B received placebo on days 1–3, followed by a loading dose of 600 mg JNJ-54175446 on day 4 (during TSD) and 150 mg JNJ-54175446 once daily through day 10. Patients in group C received placebo from day 1 through day 10.

### Safety evaluations

During the study, safety and tolerability evaluations were performed by monitoring adverse events, clinical laboratory tests (hematology, serum chemistry urinalysis and serum pregnancy test at screening and urine pregnancy tests during the treatment phase). 12-lead electrocardiograms (ECG), vital signs, and physical and neurological examination were frequently monitored and performed. Suicidal ideation and behavior was assessed using the Columbia Suicide Severity Rating Scale (C-SSRS) [59].

### Pharmacokinetics (PK)

PK blood samples were collected predose, at 1, 2, 4, 8, and 10 h post dose on day 1 and day 10 and at 24 h and 48 h after the last dose of JNJ-54175446. The maximum plasma concentration (Cmax), minimum observed plasma concentration (Cmin) and the area under the plasma concentration-time curve (AUCτ) was measured during dosing interval (τ). Just prior to the beginning or at the end of a dosing interval of any dose other than the first dose, the observed plasma concentration (Ctrough) was determined. Average plasma concentration (Cavg) was measured at steady state over the dosing interval and time to reach the maximum plasma concentration (tmax) was measured.

### Exploratory proof-of-concept related assessments

#### Procedure for acute total sleep deprivation (TSD)

The TSD procedure consisted of restructuring the individual’s diurnal rhythm and providing activities that prevented sleep and promoted wakefulness. As shown in Table 1, patients were deprived of sleep for a total of 36 h from 7:00 on day 3 to 19:30 on day 4, after which they were allowed to sleep (recovery sleep). During the TSD, study staff closely monitored the patients with the aim of gently ensuring compliance, avoiding naps and reinforcing motivation. During this period, patients were also exposed to normal ambient light (80–100 lux). During both the day preceding (07:00–19:30) and the day following (07:00–19:30) the TSD night, patients were allowed to be exposed to direct daylight for a maximum of 1 h. To enhance the effects of light, patients undergoing TSD were concurrently administered light therapy at 03:00 during and 08:00 following the sleep deprivation night [43]. The Philips HF3419 EnergyUp EnergyLight bright daylight lamp was used, exposing patients to 10,000-lux for 30 min during each administration.

#### Mood-related outcomes

Symptoms of depression were evaluated using the structured interviews, assessments, patient reported measures, and self-reported instruments outlined below. As antagonism of P2X7Rs was shown to reverse stress-induced anhedonia in preclinical studies, the Probabilistic Instrumental Learning Task (PILT) and the Snaith-Hamilton Pleasure Scale (SHAPS) were applied to assess hedonic tone. Table 2 summarizes the exact time points at which the evaluations took place.

**Table 2:** Depression questionnaire total scores (mean [SD]) per treatment group (group A JNJ-54175446, group B JNJ-54175446 post TSD, group C placebo) at different time points.

Group A (JNJ-54175446 pre and post TSD)					
Time point	HDRS17	QIDS-SR			POMS
Day-1	20.0 [4.03]	13.0 [3.98]			38.88 [16.15]
Start TSD					
Day 3	NP	10.7 [4.36]	29.00 [16.43]		
Recovery sleep					
Day 5	NP	6.7 [5.37]	15.88 [17.72]		
Day 10	14.7 [4.94]	8.7 [5.24]	17.79 [17.51]		
Follow Up	13.8 [5.01]	8.7 [5.07]	NP		
**Group B (placebo pre- TSD and JNJ-54175446 post TSD)**					
Day-1	18.1 [4.38]	12.2 [4.63]		34.40 [15.91]	
Start TSD					
Day 3	NP	10.4 [4.82]		29.08 [17.24]	
Recovery sleep					
Day 5	NP	5.9 [5.45]		12.92 [20.19]	
Day 10	13.2 [5.16]	7.3 [5.24]		17.50 [20.64]	
Follow Up	12.3 [5.63]	7.1 [5.45]		NP	
**Group C (Placebo)**					
Day-1	18.9 [4.04]	10.8 [3.52]		32.4 [15.73]	
Start TSD					
Day 3	NP	8.8 [3.91]		24.9 [14.87]	
Recovery sleep					
Day 5	NP	4.2 [4.02]		9.9 [14.81]	
Day 10	13.8 [5.49]	5.9 [3.76]		10.8 [13.04]	
Follow Up	12.9 [5.73]	5.1 [4.35]		NP	

For the depression questionnaires, the only observed significant effects were caused by TSD.

*NP* not performed, *TSD* total sleep deprivation, *HDRS17* Hamilton Depression Rating Scale, 17 items, *QIDS-SR* Self-rated Quick Inventory of Depressive Symptoms, *POMS* Profile of Mood States.

##### Hamilton depression rating scale, 17 items (HDRS17)

The HDRS17 is a clinician-administered rating scale designed to assess the severity of symptoms in MDD patients. Each of the 17 items was rated by the clinician on either a 3- or a 5-point scale; higher scores indicated a worse outcome.

##### Inventory of depressive symptoms, clinician rated 30-items (IDS-C30)

The IDS-C30 [60] is a clinician reported measure designed to assess the severity of depressive symptoms, which assesses all the criterion symptom domains designated by the DSM-IV and DSM-5.

##### The self-rated quick inventory of depressive symptoms-10/16 (QIDS-SR10/16)

The QIDS-SR10/16 is a subjective measure to assess the severity of depressive symptoms over a 1-week recall period (QIDS-SR16), or within a 2 h recall period (QIDS-SR10). The QIDS-SR16 was administered at screening, baseline, day 3, day 10 and at follow up and the QIDS-SR10 was administered in the morning and evening during the TSD period on day 4 and 5.

##### Profile of mood states brief form (POMS)

The POMS [61] was applied to measure positive and negative affective states, by evaluating positive and negative mood descriptors. The POMS was administered at baseline, day 3, in the morning and evening during the TSD period on day 4 and 5, on day 10 and at follow up.

##### Snaith-Hamilton pleasure scale (SHAPS)

The SHAPS [62] is a self-report instrument which assesses hedonic capacity. Each of the SHAPS items has a set of four response categories: Strongly Disagree, Disagree, Agree and Strongly Agree. A higher SHAPS total score indicates higher levels of present state of anhedonia. The SHAPS was administered at baseline and on days 3 and 4 (before and after TSD), 5 and 10.

### The probabilistic instrumental learning task (PILT)

The PILT [63] allows the objective assessment of a participant’s propensity to modulate behavior as a function of reward, and was administered at baseline and on days 3 and 4 (before and after TSD), 5 and 10. In each trial, one of two possible pairs of symbols was presented. One pair of symbols was associated with wins, with the “correct” symbol in this pair having a probability of winning money (0.2 euro) on 70% of trials when it was chosen, and winning nothing on the remaining 30% of trials, and the “incorrect” symbol having a probability of winning nothing in 70% of trials when it was chosen, and winning money in the remaining 30% of trials. The other pair of symbols was associated with losses, with the “correct” symbol having a probability of losing nothing on 70% of trials in which it was chosen and losing money on the remaining 30% of trials, and the “incorrect” symbol having a probability of losing money on 70% of chosen trials and losing nothing on the remaining 30% of trials. Patients first performed a shortened, 10 trial familiarization version of the PILT. Each session of the PILT task involved two, 60 trial (30 win trials, 30 loss trials) runs with each run containing a different set of 4 symbols. Patients began the task with a fixed small amount of money. On each trial, patients were randomly presented with a pair of symbols on a display screen for 4 s, with each symbol randomly positioned either to the left or the right of a central fixation cross. Patients were instructed to choose between the two symbols in order to maximize payoffs. Once a choice was made, the associated outcome was presented on the display screen. In order to maximize payoffs, patients had to use the outcome feedback to gradually learn the symbol-outcome associations over time, such that they consistently chose the symbol with the high-probability win and avoided the symbol with the high-probability loss. Outcome measures included “total amount lost”, “total amount won” and percentage of choices identical to the preceding one (“choice consistency”). “Total amount lost” measured the amount of money lost over the course of the task, which gives a rough estimate of learning performance for losses (i.e., a lower score is a better outcome). “Total amount won” was a measure of the amount won over the course of the task and gives a rough estimate of learning performance for wins (i.e., a higher score is a better outcome). “Choice consistency” is the proportion of trials in which the patient chose the same option for wins/loss trials as opposed to switching choice and is a measure of choice confidence verifying patients have learned the concept behind the test.

### Interleukin-1-beta (IL-1β)

IL-1β in peripheral blood was analyzed on day 1 (predose, 2 h and 8 h post dose), on day 2 (24 h after the initial dose of JNJ-54175446), and on day 4 during TSD (predose, 2 h and 8 h post dose). Venous blood samples were taken (TruCulture) to obtain peripheral white blood cells (WBCs) for ex-vivo stimulation using LPS/BzATP to stimulate IL-1β release. At each sampling, 1 mL blood was collected in each of two TruCulture tubes containing 100 ng/mL LPS (cat#782-001087, Myriad RBM, Austin, Texas,USA). The tubes were incubated at 37 °C for 1 h in dryblock heaters followed by addition of 2′3′-BzATP (NuBlocks, Oceanside, California, USA) to one of the tubes to a final concentration of 1 mM (using 100× stock solution). The contents of both the tubes were mixed by inversion and incubated for an additional 1.5 h. The tubes were then centrifuged (1000 *rpm*, 10 min at room temperature) and supernatants were collected in a new tube, frozen and were later analyzed to determine IL-1β concentration (V-plex Proinflammatory Panel 1 Human Kit, cat#K15049D, MesoScale Discovery, Rockville, Maryland, USA). The concentration of IL-1β in the tube that contained only LPS was subtracted from the concentration of IL-1β in the tube that had both LPS and BzATP (LPS+BzATP) to quantify the P2X7-dependent IL-1β release. TruCulture tubes containing only blood served as negative controls for circulating IL-1β levels (NULL tube, cat#782-001086, Myriad RBM).

### Analysis

#### Sample size calculation

This safety and tolerability study involved TSD as an exploratory proof-of-concept intervention to induce acute mood improvement and was preceded and/or followed by JNJ-54175446 and/or placebo. Hence, it was impossible to make any plausible assumptions of the effect sizes and/or the interactions between sleep deprivation, treatment (duration) and mood changes. Therefore, formal statistical calculation of sample size was not appropriate and was not performed. Generally speaking, a sample size of 60 patients or more represents the customary size employed in early development studies designed to allow clinical assessment of safety and tolerability and is sufficient to demonstrate clinical mood elevating effects of sleep deprivation and/or antidepressant medication [64], and to allow accurate assessment of the pharmacokinetic profile of novel compounds.

#### Statistics

Since the study was not primarily powered to assess the pharmacodynamic effects of JNJ-54175446, descriptive statistics were performed for HDRS17, QIDS-SR POMS, PILT and SHAPS, with mean ± SD values and changes from baseline being summarized by treatment group at the respective scheduled time points per instrument. Data were presented graphically for individual subject values or as mean ± SE values over time.

In addition to the descriptive statistics, for the HDRS17 total score a mixed-effects model using repeated measures was performed for change from baseline (day -1) to day 10. The model included baseline score as covariate, and antidepressant treatment status (treatment naïve or SSRI treatment), day, treatment and day-by-treatment interaction as fixed effects, and a random subject effect.

In blood, both in vivo and ex-vivo, mean values and mean changes from baseline in IL-1β levels were determined and compared to placebo.

Following review and interpretation of the exploratory results, a pattern became apparent for the effects of JNJ-54175446 on hedonic tone during the TSD period: all patients receiving JNJ-54175446 before the recovery night (which were both groups A and B, as group B received their loading dose on the last day of TSD) did not demonstrate changes in PILT ‘total amount lost’, whereas placebo group C demonstrated a clear improvement following recovery sleep. Therefore, specific additional exploration of hedonic tone as assessed with the SHAPS and the PILT total amount lost was warranted. To this end a post-hoc analysis was performed to further assess the effect of JNJ-54175446 compared to placebo on TSD induced decrease of the SHAPS total score and PILT total amount lost, between day 3 and day 5 using an unpaired *t*-test with Welch’s correction.

## Results

**Fig. 1:**
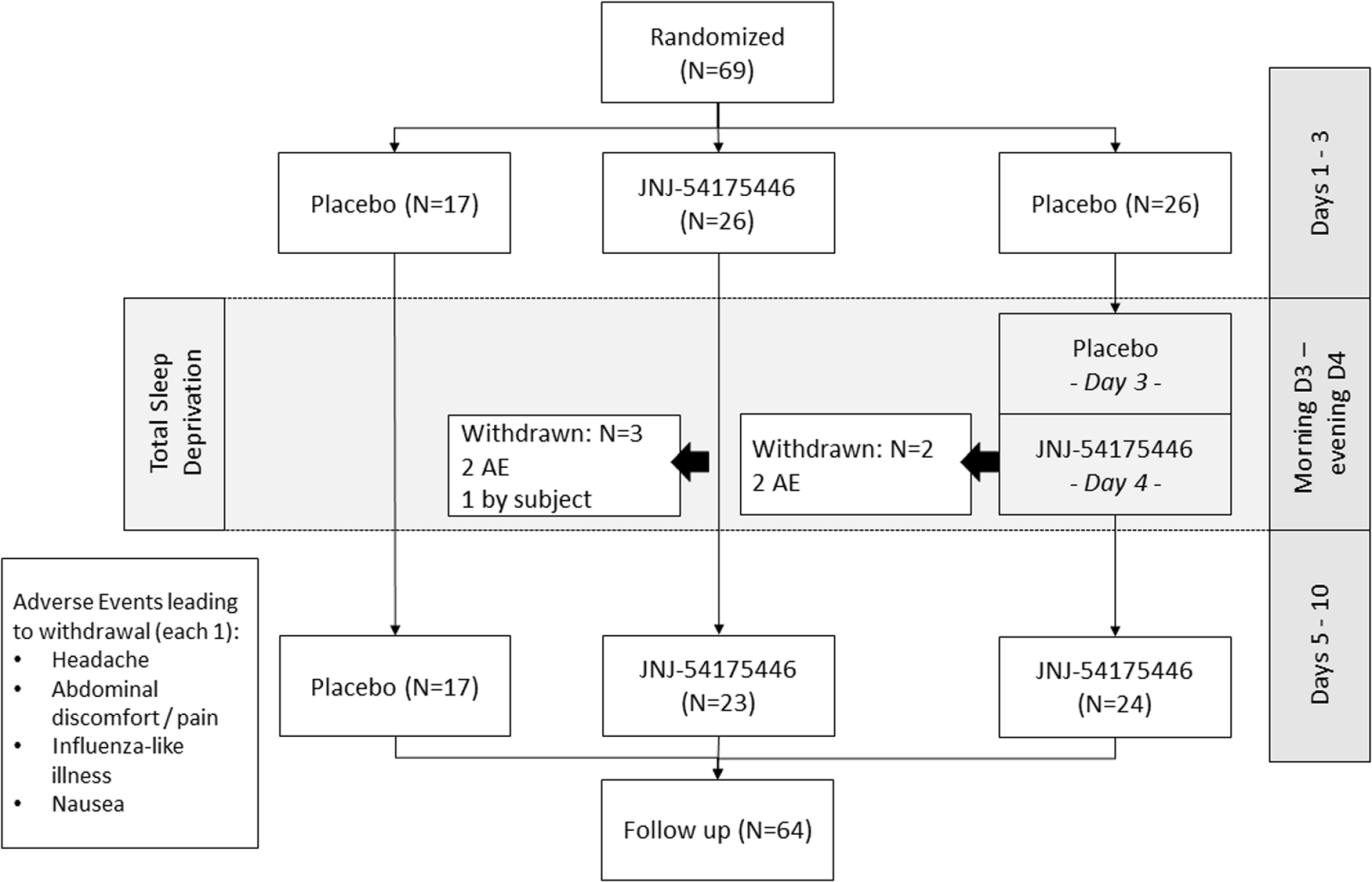
Disposition schedule. This schedule describes how many patients were assigned to each group and completed the study.

Sixty-nine patients received at least one dose of JNJ-54175446, and a total of 64 patients completed the study. The study disposition schedule is shown in Fig. 1. In the JNJ-54175446 early start group A, 23 (88.5%) of 26 patients completed the treatment phase and three patients were withdrawn from the study: two patients due to adverse events (headache and abdominal discomfort) and one patient withdrew consent. In the JNJ-54175446 late start group B, 24 (92.3%) of 26 patients completed the treatment phase and two patients were withdrawn from the study due to TEAEs (influenza-like illness and nausea and chills). In all cases who were withdrawn due to an adverse event, the study drug was discontinued and the treatment emergent symptoms resolved. In the placebo group C, all 17 patients (100%) completed the treatment phase. In total, 62.3% of patients were female and 37.7% of patients were male. The mean age was 38.6 years (range: 18–62 years) and the mean body mass index (BMI) was 25.13 kg/m^2^ (range: 19.3–32.1 kg/m^2^). There was no marked difference in demographic and baseline characteristics across the treatment groups. The average IDS-C30 total score (mean [SD]) at baseline was 40.4 [1.29] for group A, 39.1 [1.11] for group B and 37.2 [1.28] for group C, respectively. Of all included patients, 88.4% were medication-free while 11.6% were receiving a SSRI (randomization was stratified for subjects who were treatment naïve or subjects treated with SSRI monotherapy).

### Adverse events

JNJ-54175446 was well-tolerated and all adverse events were mild to moderate. There were no deaths, serious AEs, or persistent AEs reported in this study. In group A (JNJ-54175446 early start) two TEAEs, headache and abdominal pain lead to study discontinuation. Both were classified as severe and considered by the investigator as possibly related to JNJ-54175446. In group B (JNJ-54175446 late start) the two TEAEs that lead to study discontinuation were an influenza-like illness starting at day 1 that was considered by the investigator not related to JNJ-54175446 and moderate intensity chills and nausea which were considered to be very likely related to JNJ-54175446. It should however be noted that the patient was suffering from nausea before the administration was started.

Adjusting for only those patients who received JNJ-54175446, the following TEAEs were the most reported; headache (25.0%), nausea (11.5%), dysgeusia (5.8%) and vomiting (5.8%). No clinically significant changes were observed in laboratory evaluations. Changes from baseline in vital signs and ECG parameters were small and not considered clinically relevant.

### Pharmacokinetics

Pharmacokinetic results are shown in Table 3. Mean plasma concentrations following a single dose of 600 mg JNJ -54175446 showed a similar profile in both group A (JNJ-54175446 early start) and B (JNJ-54175446 late start), with peak concentrations being reached at 8 h post dose followed by a slight decline in concentration up to 24 h post dose. On day 10, mean plasma concentrations following multiple doses of 150 mg JNJ-54175446 showed a slight increase from the predose concentrations on day 10, reaching a peak at 4 h post dose. The mean accumulation ratios were similar for both treatment groups with AUCτ values on day 10 approximately 1.9-fold higher than the AUCτ values on day 1 or day 4 for groups A (JNJ-54175446 early start) and B (JNJ-54175446 late start), respectively. After reaching a peak, the mean plasma concentrations declined slowly at a similar rate following both treatment groups until the last sample taken at 168 h post dose. Mean half-life (t_1/2term_) was similar for both treatment groups on day 10 (50.2 h following group A and 55.5 h following group B). All patients still had quantifiable JNJ-54175446 plasma concentrations at 168 h post dose.

**Table 3:** Pharmacokinetic results of JNJ-54175446 (Mean [SD]; tmax, median [range]) after administration of JNJ-54175446 at 600 mg on day 1, followed by 150 mg JNJ-54175446 qd on days 2 through 10 (group A) and after placebo on days 1 to 3, followed by 600 mg JNJ-54175446 on day 4, followed by 150 mg JNJ-54175446 qd on days 5 through 10 (group B).

Day	Parameter	Group A	Group B
Day 1	n	26	NP
	C_max_ (ng/mL)	1181 (394)	NP
	C_min_ (ng/mL)	BQL	NP
	T_max_ (h)	7.98 (3.93–23.33)	NP
	AUC_t_ (ng.h/mL)	21107 (6841)	NP
Day 4	n	24^a^	22^b^
	C_max_ (ng/mL)	1630 (593)	1225 (340)
	C_min_ (ng/mL)	1269 (433)	BQL
	T_max_ (h)	4.05 (0.00–23.33)	6.09 (2.15–22.33)
	AUC_t_ (ng.h/mL)	34512 (10301)	22383 (5909)
Day 10	n	23^c^	24^d^
	C_max_ (ng/mL)	1932 (664)	1894 (365)
	C_min_ (ng/mL)	1417 (510)	1368 (297)
	C_avg_ (ng/mL)	1671 (591)	1615 (315)
	T_max_ (h)	4.00 (2.00–10.00)	4.02 (3.73–10.00)
	AUC_t_ (ng.h/mL)	39905 (14069)	38504 (7556)
	AUC_last_ (ng.h/mL)	158406 (74147)	158953 (56655)
	AUC_∞_ (ng.h/mL)	174933 (88211)	167185 (55751)
	T_1/2term_ (h)	50.2 (17.8)	55.5 (22.4)
	Peak/trough ratio	1.27 (0.133)	1.33 (0.171)

*BQL* below quantification limit (<2.50 ng/mL), *Cmax* maximum plasma concentration, *Cmin* minimum observed plasma concentration, *Cavg* Average plasma concentration, *AUCt* area under the plasma concentration-time curve, *AUClast* area under the analyte concentration, *AUC∞* area under the plasma concentration-time curve from time 0 to infinity, *T1/2term* terminal half-life.

^a^*n* = 23 for AUCt.

^b^*n* = 24 for Cmin.

^c^*n* = 20 for AUC∞.

^d^*n* = 23 for AUClast and T1/2term; *n* = 22 for RA.AUC; *n* = 19 for AUC∞.

### Exploratory proof-of-concept related assessments

Table 2 provides an overview of the total scores for all depression questionnaires observed at different time points during the trial.

#### Mood symptoms

##### Hamilton depression rating scale (HDRS)

At baseline (day-1), the HDRS17 total score (mean [SD] was 20.0 [4.03] for group A (JNJ-54175446 early start); 18.1 [4.38] for group B (JNJ-54175446 late start); and 18.9 [4.04] for group C (placebo), respectively.

Effects of TSD per average HDRS17 total score [SD], measured as change from baseline (day -1) on day 4, were −2.3 (3.82) in group C (placebo), somewhat larger (−3.9 (2.97)) in group A, and slightly smaller (−1.5 (3.33)) in group B. Although on day 4 a numerically larger proportion of patients demonstrated a ≥ 25% improvement on the HDRS17 total score in group A [9/25 (36%)] compared with group C [5/27 (29%)] or group B [5/25 (20%)], these differences were not significant (*X*^2^ = 1.588; *p* = 0.452).

No significant differences (mean change [SD]) on day 10 compared with baseline were observed in any treatment group versus placebo: Group A −4.8 [4.69] *p* = 0.663; Group B −5.1 [4.26] *p* = 0.481 and Group C −5.1 [5.71]), respectively (Supplemental Fig. 1).

### The self-rated quick inventory of depressive symptoms (QIDS-SR)

The average Quick Inventory of Depressive Symptoms (QIDS-SR) total score (mean [SD]) at baseline (day -1) was 13.0 [3.98] for group A (JNJ-54175446 early start); 12.2 [4.63] for group B (placebo pre TSD and JNJ-54175446 late start) and 10.8 [3.52] for group C (placebo) (supplemental Fig. 2).

TSD decreased the average QIDS-SR10 total score with a maximal decrease observed in the morning of study day 5 (i.e., after recovery sleep). Mean values [SD] for the QIDS-SR10 total score, assessed on Day 4 and Day 5 (between 2 and 8 h postdose on both study days) decreased from 8.2 to 4.0 [−4.2] for subjects in group C, versus 9.2–6.9 [−2.3] and 10.2–5.8 [−4.4] for subjects in the JNJ-54175446 treated groups A and B, respectively. The effects of TSD were sustained until the last day of treatment (day 10) on which the mean total score (SD) was 8.7 [5.24] for group A; 7.3 [5.24] for group B and 5.9 [3.76] for group C.

### Profile of mood scales (POMS)

The Profile of Mood Scales (POMS) total score (mean [SD]) at baseline (day -1) was 38.9 [16.2] for group A (JNJ-54175446 early start); 34.4 [15.9] for group B (JNJ-54175446 late start) and 32.4 [15.7] for group C (placebo).

The average POMS total score (SD) decreased following TSD and recovery sleep from day 4 to day 5 in group A from 33.9 (17.8) to 15.9 (17.7), in group B from 35.6 (16.9) to 13.0 (20.2) and in group C from 31.1 (10.8) to 9.9 (14.8). Reductions were observed for the individual sub-scales: tension/anxiety, depression/dejection fatigue/inertia, anger/hostility and confusion/bewilderment, but an increase in was found for the positive mood descriptor vigor/activity. These changes were consistent across all treatment groups, indicating that there was no clear interaction between TSD effect and JNJ-54175446 treatment. The average POMS total score (SD) on day 10 was 17.8 (17.5) for group A; 17.5 (20.6) for group B and 10.8 (13.0) for group C.

#### Hedonic capacity

Table 4 provides an overview of the total scores for all anhedonia measurements observed at different time points during the trial.

**Table 4:** Anhedonia assessments total scores (mean [SD]) per treatment group at different time points.

Group A (JNJ-54175446 pre and post TSD)					
Time point	SHAPS	PILT total amount won	PILT choice consistency wins	PILT total amount lost	PILT choice consistency losses
Day-1	8.6 [2.26]	17.72 [2.15]	0.79 [0.16]	14.22 [1.86]	0.61 [0.12]
Start TSD					
Day 3	7.5 [3.31]	16.71 [2.54]	0.81 [0.18]	13.07 [2.17]	0.66 [0.17]
Recovery sleep					
Day 5	7.4 [4.05]	17.38 [3.01]	0.83 [0.17]	12.84 [2.29]	0.68 [0.18]
Day 10	6.4 [3.96]	18.25 [2.78]	0.87 [0.14]	12.00 [1.67]	0.71 [0.17]
**Group B (placebo pre- TSD and JNJ-54175446 post TSD)**					
Day-1	8.15 [3.50]	16.82[2.22]	0.72 [0.18]	14.54 [1.93]	0.60 [0.13]
Start TSD					
Day 3	7.28 [3.58]	17.1	0.77	14.49	0.61
		[2.77]	[0.18]	[2.09]	[0.16]
Recovery sleep					
Day 5	7.33 [4.28]	17.20 [2.42]	0.77 [0.20]	13.94 [2.50]	0.65 [0.17]
Day 10	5.71 [4.42]	18.33 [2.69]	0.83 [0.17]	12.36 [2.43]	0.71 [0.18]
**Group C (Placebo)**					
Day-1	8.2 [2.68]	15.29 [2.13]	0.70 [0.17]	14.22 [1.75]	0.60 [0.08]
Start TSD					
Day 3	6.9 [2.97]	16.24 [2.84]	0.79 [0.19]	14.25 [2.10]	0.65 [0.14]
Recovery sleep					
Day 5	4.8 [3.17]	17.51 [3.02]	0.87 [0.17]	12.67 [2.00]	0.70 [0.17]
Day 10	4.6 [4.29]	16.92 [2.96]	0.85 [0.15]	13.02 [1.65]	0.71 [0.14]

_*TSD* total sleep deprivation, *SHAPS* Snaith-Hamilton Pleasure Scale, *PILT* Probabilistic Instrumental Learning Task._

### Snaith-Hamilton pleasure scale (SHAPS)

**Fig. 2:**
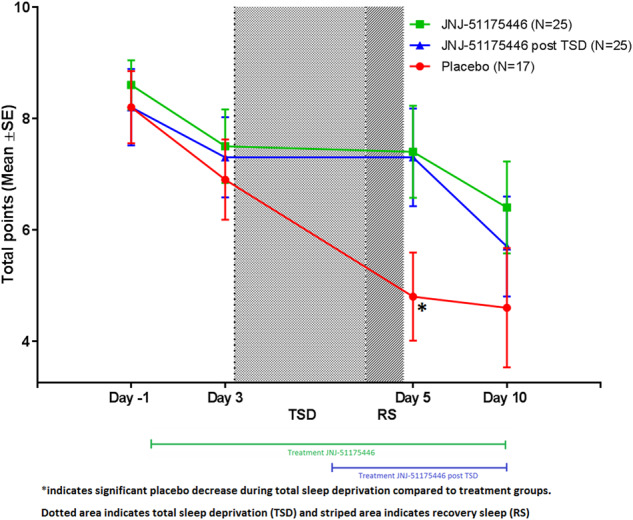
Snaith-Hamilton Pleasure Scale (SHAPS). This figure displays the Snaith-Hamilton Pleasure Scale (SHAPS) Total Score Mean [SD] over time for group A (green square), group B (blue triangle) and group C (red circle).

The Snaith-Hamilton Pleasure Scale (SHAPS) total score (mean [SD]) at baseline (day -1) was 8.6 [2.26] for group A (JNJ-54175446 starting before TSD); 8.15 [3.50] for group B (placebo pre-TSD and JNJ-54175446 starting with loading dose on recovery night) and 8.2 [2.68] for group C (all-placebo) (Fig. 2). For the SHAPS a higher score indicates more severe anhedonia.

The TSD-induced decrease of total SHAPS score, measured as the change from day 3 to day 5, was significantly attenuated in group A (JNJ-54175446 early start) (0.0 [3.64]) compared to group C (placebo) (−2.0 [2.58], *p* = 0.049). There was a similar trend between groups B (−0.24 [3.03]) and C (placebo) (−2.0 [2.58]) but this difference did not reach statistical significance (*p* = 0.055). While average total SHAPS scores were significantly reduced from day -1 to day 10 (*p* = 0.005) by −2.5 [3.98] in Group A [*F*(4,92) = 2.921; *p* = 0.025), no significant reduction in SHAPS scores was observed on day 5 after TSD (−1.3 [3.55]). Similarly, in group B [F(4,94) = 3.811; *p* = 0.006], a significant reduction in SHAPS scores was observed on day 10 (−2.2 [3.92]; *p* = 0.005), but not on day 5 (−1.1 [3.37]).

### The probabilistic instrumental learning task (PILT)

**Fig. 3:**
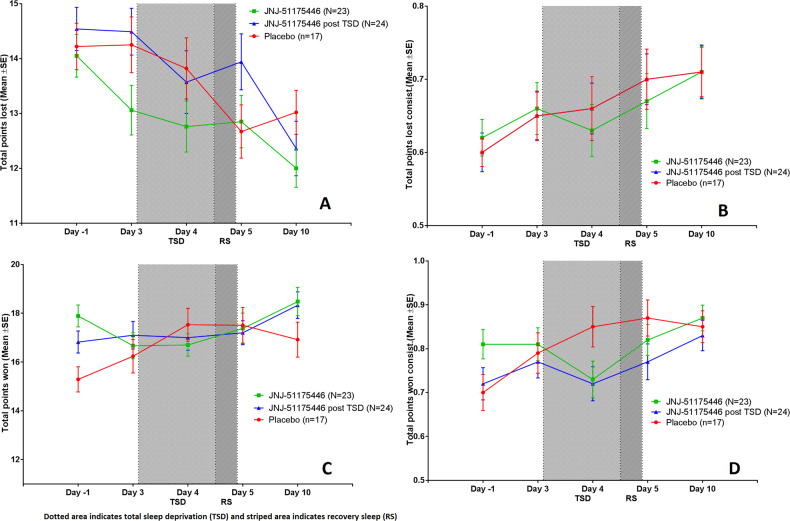
Probabilistic Instrumental Learning Task (PILT). This figure displays the Mean [SD] over time for group A (green square), group B (blue triangle) and group C (red circle) for the Probabilistic Instrumental Learning Task (PILT) for total points loss (**A**), choice consistency for loss trials (**B**), total points won (**C**) and choice consistency for win trials (**D**).

The results of the PILT are shown in Table 4 and Fig. 3. In the all-placebo group C, TSD and repeated testing had clear effects on the various outcome parameters of the PILT. “Total amount lost” decreased from 14.3 items on day 3 (before TSD) to 12.7 on day 5 (after recovery night). “Total amount won” increased from 16.2 on day 3 to 17.5 on day 5. Compared to the clear TSD-effects during placebo, sleep deprivation may have had somewhat less of an effect on PILT in the groups treated with JNJ-54175446 (supplemental Table 1). Although differences were statistically not significant, they trended in the same direction as the SHAPS. Group A received JNJ-54175446 from day 1 and group B from day 4, so both had active P2X7 inhibition during the recovery night of day 4/5. In group A with the longest drug exposure, the “total amount lost” hardly decreased from 13.1 on day 3 to 12.8 on day 5. This reduction was non-significantly (*p* = 0.0916) smaller than the 1.59 item reduction in all-placebo group C. Group B, which had received a loading dose of JNJ-54175446 just prior to the recovery night, showed a somewhat larger decrease in “total amount lost” of 0.54 items, from 14.5 on day 3 to 13.9 on day 5 (*p* = 0.1977 versus placebo group C). No clear drug effects were observed on “total amount won”. Both groups A and B that were treated with JNJ-54175446 showed small increases from 16.7-17.1 items on day 3, to 17.4–17.2 on day 5. A somewhat larger increase of 1.27 items was found in the all-placebo group C, but the differences were not significant (*p* = 0.5979 and *p* = 0.3339, resp.). After TSD, “total points lost” in placebo group C slightly increased again from 12.7 on day 5 to 13.0 on day 10. “Total points won” slightly decreased from 17.5 on day 5 to 16.9 on day 10. In group A (JNJ-54175446 pre- and post TSD), “total points lost” decreased slightly from 12.8 on day 5 to 12.0 on day 10. “Total points lost” in group B (placebo pre-TSD and JNJ-54175446 post-TSD) decreased from 13.9 on day 5 to 12.4 on day 10. Finally, “total points won” slightly increased from 17.4 on day 5 to 18.3 on day 10 in group A (JNJ-54175446 pre- and post TSD) and from 17.2 on day 5 to 18.3 on day 10 in group B (placebo pre-TSD and JNJ-54175446 post-TSD). These post TSD changes were not statistically significant compared to placebo.

#### Interleukin-1-beta (IL-1β)

Compared to placebo, no differences were observed in either treatment group for plasma IL-1β at the various assessed time points. TSD however increased levels of ex-vivo stimulated IL-1β release on day 4. These elevations were markedly attenuated compared to baseline (day -1) in the early start JNJ-54175446 (pre-TSD) treatment group A (JNJ-54175446 early start) and remained attenuated through to end of study day 10. Levels of ex-vivo stimulated IL-1β release in group B (JNJ-54175446 late start) were comparable on day 1 and day 4, but attenuated at day 10. A detailed description of the IL-1β and ex-vivo IL-1β stimulation results can be found in the supplemental material (supplemental Figs. 3, 4 and 5).

## Discussion

This was the first study to investigate the safety and pharmacokinetics of the CNS penetrant and selective P2X7 receptor antagonist JNJ-54175446 in MDD, and to explore its potential effects on mood-related phenomena such as reward propensity and changes in peripheral cytokine production following TSD. Although healthy subjects had received single doses of JNJ-54175446 up to 600 mg [39] and multiple doses up to 450 mg for 10 days [40] in previous studies, this was the first time JNJ-54175446 was administered to MDD patients. A single dose of 600 mg JNJ-54175446, followed by once daily doses of 150 mg of JNJ-54175446 was safe and well tolerated by MDD patients. Adjusting for only those patients who received JNJ-54175446, headache, nausea, dysgeusia and vomiting were the commonly reported AEs. The nausea, dysgeusia and vomiting mainly occurred shortly after administration of JNJ-54175446 which was provided as an oral suspension. To compare, the most reported AEs in healthy volunteers in an earlier study with JNJ-54175446 were headache, fatigue and backpain. Notably, these healthy volunteers underwent a lumbar puncture which could explain the backpain [40]. No clinically significant changes were observed in laboratory evaluations. Changes from baseline in vital signs and ECG parameters were small and not considered clinically relevant. This is also in line with results found in earlier studies performed in healthy volunteers [39]. Pharmacokinetic analysis confirmed that the pharmacokinetics in MDD patients are comparable to those found in these earlier clinical healthy volunteer studies, which could explain the similar AE profile.

TSD was applied as a behavioral challenge to investigate whether its effects on mood and mood-related symptoms could be modulated by JNJ-54175446. TSD alleviated depressive symptoms on both self-reported and clinician rated rating instruments, which lasted until the last study day (day 10). Although this again confirms TSD as an adequate behavioral challenge paradigm to induce a rapid recovery from depressive symptoms, it conflicts with pre-existing literature reporting transient mood improvement that disappears after a single post-TSD night of recovery sleep [45–49]. This made it difficult to interpret the effects of JNJ-54175446 on transient mood improvement after TSD, as our study design was based on this pre-existing literature, by assuming the mood state would return to baseline levels after recovery sleep in the placebo condition.

JNJ-54175446 demonstrated no effect on depressive symptoms when administered prior to TSD, suggesting that JNJ-54175446 does not produce rapidly-acting antidepressant effects. Furthermore, no significant effects of JNJ-54175446 were observed on mood improvement, which in all groups occurred after TSD and subsequently did not substantially change during the remainder of the study. However, the reduction in anhedonia observed (SHAPS) in the placebo group after recovery sleep was significantly attenuated by JNJ-54175446 in both the early (day 1) and late (day 4) starting groups. Since JNJ-54175446 was administered for the first time in the delayed group as a loading dose during the TSD procedure on the morning before the recovery sleep, this effect on hedonic capacity appeared to occur acutely, suggesting a stabilizing effect of JNJ-54175446 even after a single high dose on the anhedonia levels, such that they persisted during TSD. A similar pattern, although not statistically significant, was suggested by the total points lost on the PILT assessment of reward learning. This apparent mood stabilizing effect, which nevertheless blunted the beneficial effects of TSD on anhedonia, contrasts with the effects of P2X7R antagonism on stress-induced anhedonia seen in animal experiments, [28, 29] in which resolution of the deficit in drinking sucrose-water (anhedonia) was observed in mice receiving brain penetrant P2X7 antagonists. The difference between these preclinical and clinical models, which identified antidepressant-like and mood stabilizing effects, respectively, conceivably may reflect differences in drug exposure, as discussed below, and are noteworthy given our findings that the effects of JNJ-54175446 on the sensitization to subjective effects of dexamphetamine during repeated dosing in healthy subjects differed based on dose [40]. Taken together, however, these data sets suggest an involvement of P2X7R in stress-related changes in hedonic capacity.

Overall, there were no differences in levels of IL-1β in peripheral blood in response to JNJ-57417446 treatment as compared to placebo, which may reflect the multiple paths for releasing IL-1β that do not involve P2X7 receptors. However, JNJ-57417446 did decrease ex-vivo IL-1β release by LPS-stimulated peripheral white blood cells in the presence of the P2X7 receptor agonist BzATP. This provided proof of pharmacological activity in the blood, which considering the high blood-brain-barrier permeability of JNJ-57417446 is also likely to have occurred in the CNS.

Several limitations were identified in this study. We observed a protracted effect of TSD on depression severity ratings in MDD patients and, as a consequence, could not determine a clear difference between and the placebo group and patients receiving a loading dose of JNJ-54175446 prior to the TSD recovery night. Although previous investigations suggested that combining TSD with bright light therapy is effective in sustaining its antidepressant effect [43, 65, 66], the prolonged retention of antidepressant effects in the current study could be related to placebo-effects, but also to the exposure to light during TSD. Furthermore, the study, being primarily a safety and tolerability study, was not powered to demonstrate an antidepressant effect or modulation of hedonic tone, but merely to explore such potential pharmacodynamic effects. In addition, only one dose of JNJ-54175446 was studied, and the optimal dose range for mood-related efficacy in MDD was unknown during the study design. The 600 mg loading and 150 mg maintenance dose were mainly based on JNJ-54175446’s attenuating effects on dexamphetamine-induced motor improvements in healthy subjects, but may actually have been supratherapeutic for MDD. The EEG and cognitive effects of JNJ-54175446 under placebo and dextroamphetamine challenge showed some profound differences between the 50 mg dose (which achieved nearly full P2X7R occupancy in the brain in PET studies and is being tested for antidepressant efficacy in an ongoing clinical trial of MDD [67]) and higher doses (e.g., see Table 4, 5, 7 of Recourt et al. [40]). Thus, it is conceivable that lower JNJ-54175446 exposures are required for mood-related modulation compared with motor-related effects. Finally, the current trial did not enrich for patient populations with evidence of increased inflammation that may have benefited more from anti-inflammatory intervention, such as patients with elevated inflammatory markers or a somatic comorbidity. Identifying such subgroups and adding other cascade markers in future studies with JNJ-57417446 could provide more consistent results in both efficacy and pro-inflammatory mediator endpoints [68].

In conclusion, JNJ-54175446 was safe and well tolerated by MDD patients. In line with its pharmacological properties, JNJ-54175446 reduced IL-1β release from LPS-stimulated peripheral white blood cells in the presence of the P2X7R agonist BzATP. TSD (combined with light therapy) alleviated depressive symptoms on both self-reported and clinician rated rating instruments, which lasted until the end of the observation period (day 10). This is longer than previously reported in the literature, which may have prevented evaluation of any potential antidepressant effect of post-TSD administration of JNJ-54175446. However, the current study suggested that JNJ-54175446 at the relatively high exposures tested may blunt the reduction of anhedonia that occurs in MDD patients after TSD. These potential mood stabilizing effects on hedonic capacity warrant further exploration in future studies of the P2X7 antagonist mechanism.

## Supplementary information

Supplemental material
