# Supplementary material for: Characterization of the central nervous system penetrant and selective purine P2X7 receptor antagonist JNJ-54175446 in patients with major depressive disorder
Source: Transl Psychiatry. 2023 Jul 24;13:266. doi: 10.1038/s41398-023-02557-5 (PMC10363543; doi:10.1038/s41398-023-02557-5)
Supplement: Supplementary file 1 — Supplemental material [file 41398_2023_2557_MOESM1_ESM.pdf]

Supplemental table 1. Difference between Anhedonia assessments total scores (mean [SD]) before (day 3) and after (day 5) TSD per treatment group (p-values compared to placebo).

Group / Score	Group A (JNJ-54175446 pre- and post TSD)	Group B (placebo pre-TSD and JNJ-54175446 post-TSD)	Group C (placebo)
Delta SHAPS (day 3-5)	0.00 [3.78] (p= 0.0491)	-0.24 [2.53] (p=0.0546)	-2.00 [2.58]
Delta PILT total amount lost (day 3-5)	-0.21 [2.24] (p=0.0916)	-0.54 [2.40] (p=0.1977)	-1.59 [2.57]
Delta PILT choice consistency losses (day 3-5)	0.01 [0.17] (p=0.5227)	0.02 [0.21] (p=0.9833)	0.05 [0.12]
Delta PILT total amount won (day 3-5)	0.71 [2.74] (p=0.5979)	0.22 [2.00] (p=0.3339)	1.27 [4.06]
Delta PILT choice consistency (day 3-5)	0.01 [0.18] (p=0.2414)	-0.03 [0.26] (p= 0.1385)	0.08 [0.15]

Supplemental figure 1. Hamilton Depression Rating Scale, 17 items (HDRS17) Total Score: Means ±SE Over Time

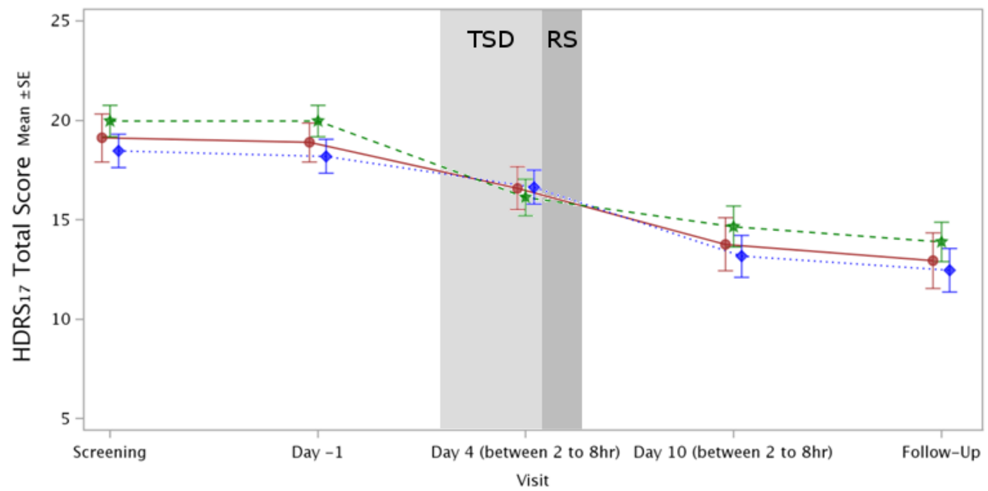

Number of Subjects					
Placebo (N=17)	17	17	17	17	17
JNJ-54175446 (N=26)	26	26	25	23	25
JNJ-54175446 post-TSD (N=26)	26	26	25	24	26

Placebo (N=17) JNJ-54175446 (N=26) JNJ-54175446 post-TSD (N=26)

Grey area indicates total sleep deprivation (TSD) and dark grey area indicates recovery sleep (RS)

Supplemental figure 2 Self-rated Quick Inventory of Depressive Symptoms-10 (QIDS-SR10) Total Score: Means ±SE Over Time

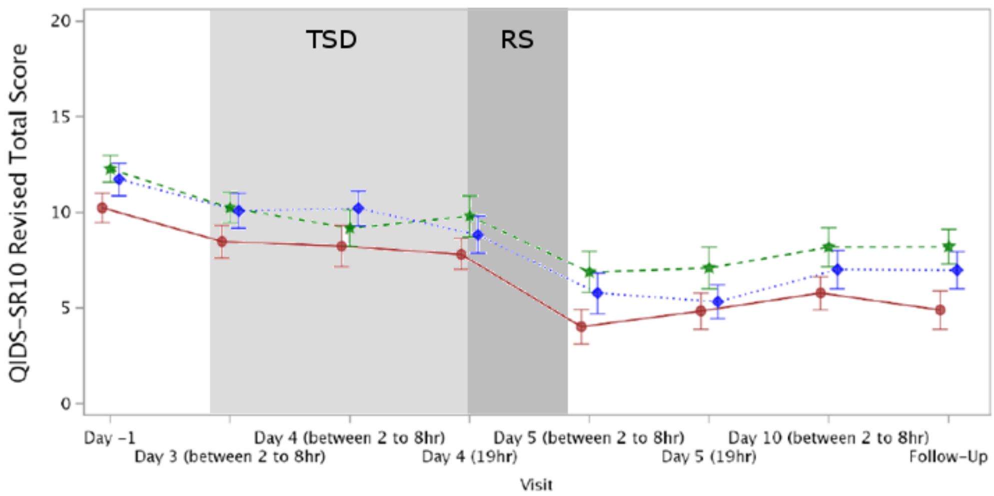

Number of Subjects								
Placebo (N=17)	17	17	17	16	17	17	17	17
JNJ-54175446 (N=26)	26	25	24	24	24	23	23	25
JNJ-54175446 post-TSD (N=26)	26	25	25	23	25	25	24	26

Placebo (N=17) JNJ-54175446 (N=26) JNJ-54175446 post-TSD (N=26)

Grey area indicates total sleep deprivation (TSD) and dark grey area indicates recovery sleep (RS)

Supplemental figure 3. Interleukin (IL)-1β concentrations at various time points

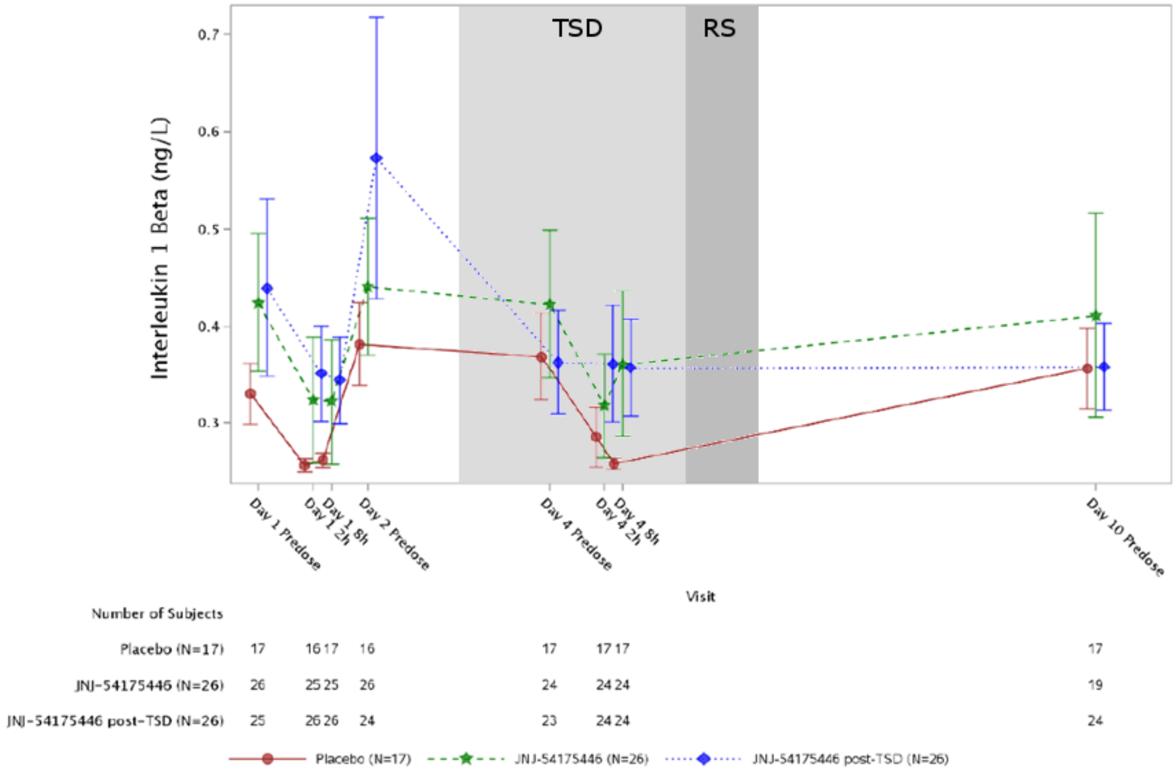

Grey area indicates total sleep deprivation (TSD) and dark grey area indicates recovery sleep (RS)

Mean (±SE) values of IL-1β in blood declined in all groups independent of treatment with JNJ-54175446 from day 1 predose values: (group A: [0.4240 ± 0.07137 ng/L], group B: [0.4392 ± 0.09134 ng/L] and group C: placebo [0.3299 ± 0.0317 ng/L]), to day 1, 2 hours and day 1 8 hours postdose values: (group A [0.3217 ± 0.06460 ng/L], group B [0.3439 ± 0.04510 ng/L] and group C [0.2609 ± 0.00732 ng/L]), returning to near baseline levels after 24 hours (day 2). The expected increase in plasma concentrations of the pro-inflammatory mediator IL-1β post sleep deprivation, was not observed on day 4:(group A [0.4223 ± 0.07660 ng/L], group B [0.3627 ± 0.05383 ng/L] and group C [0.3682 ± 0.04501 ng/L]), as compared to the predose timepoint (group A [0.4409 ± 0.07032 ng/L], group B [0.5729 ± 0.14509 ng/L] and group C[0.3813 ± 0.04232 ng/L]). Overall, no differences in the response to JNJ-574175446 treatment versus placebo were observed (Supplemental figure 3).

Supplemental figure 4. Interleukin (IL)-1β concentrations released on LPS stimulation at various time points

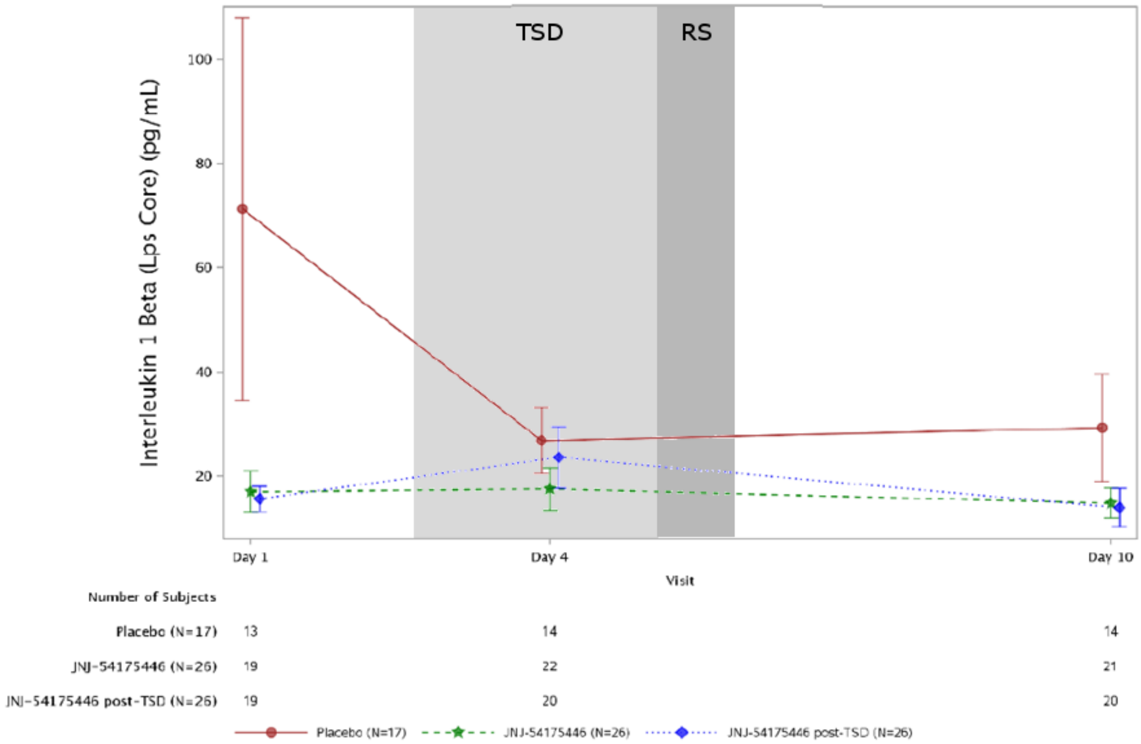

Grey area indicates total sleep deprivation (TSD) and dark grey area indicates recovery sleep (RS)

Mean (±SE) values of IL-1β released on LPS stimulation of peripheral white blood cells from patients showed a small increase in IL-1β levels from day 1 (group A [17.014 ± 3.9575 pg/mL], group B [15.620 ± 2.4045 pg/mL]) to day 4 predose (ie, following day 3 to day 4 sleep deprivation) in group B, that was not observed in group A (group A [17.539 ± 4.0772 pg/mL], group B [23.578 ± 5.8910 pg/mL]). IL-1β levels returned to near baseline levels by day 10 (group A [14.822 ± 2.8725 pg/mL], group B [13.953 ± 63.702 pg/mL]). High baseline levels in the placebo group (group C: [71.258 ± 36.655 pg/mL], day 4 [26.835 ± 6.3234 pg/mL], day 10 [29.251 ± 10.240 pg/mL],) reflect patient variability (supplemental figure 4).

Supplemental figure 5. Interleukin (IL)-1β concentrations in the presence of the P2X receptor agonist BzATP at various time points

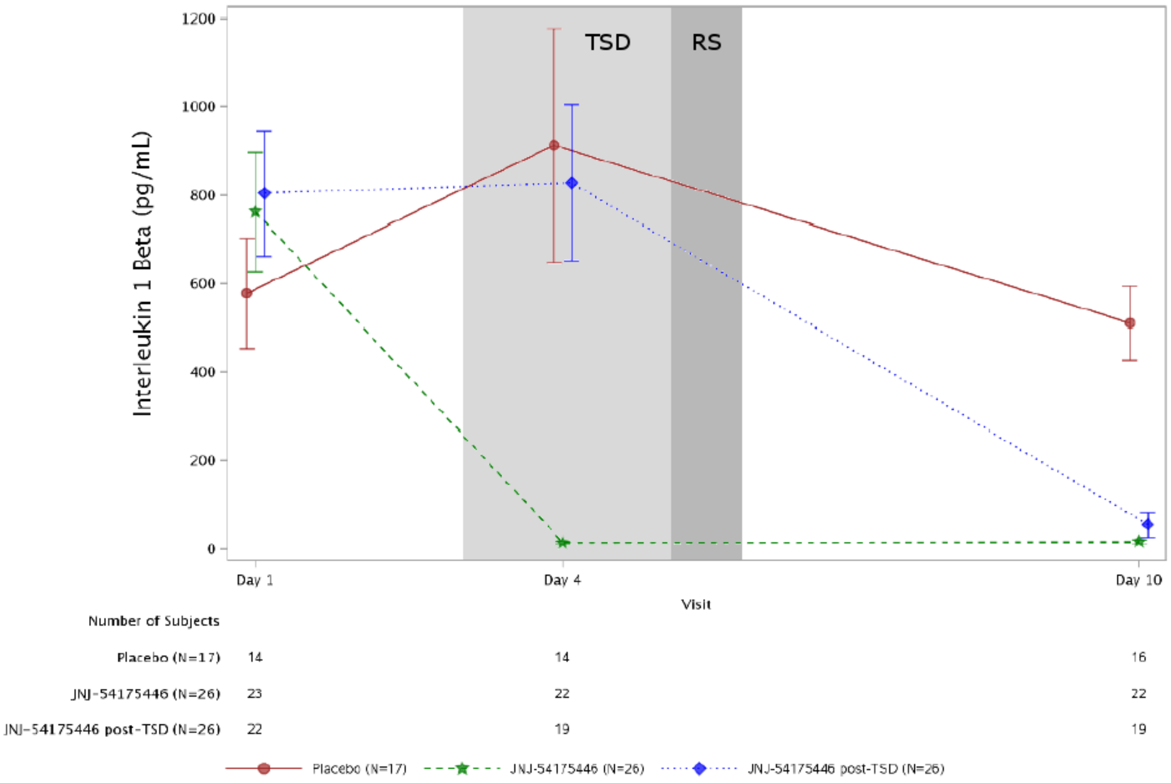

Grey area indicates total sleep deprivation (TSD) and dark grey area indicates recovery sleep (RS)

Levels of IL-1β production by LPS stimulated peripheral white blood cells isolated from patients following total sleep deprivation (day 4) were enhanced in the presence of the P2X receptor agonist BzATP, returning to near baseline levels by day 10 (group C: day 1 [577.952 ±124.4223 pg/mL], day 4 [913.934 ±265.0952 pg/mL], day 10 [510.265 ±84.0070 pg/mL]). In contrast, increased levels of IL-1β production following total sleep deprivation (day 4) were markedly attenuated in the JNJ-54175446 (pre-TSD) treatment group A and remained attenuated through to end of study day 10 (group A: (day 1 [762.622 ± 135.3801 pg/mL], day 4 [14.430 ± 2.6723 pg/mL], day 10 [16.601 ± 4.0257 pg/mL]). Levels of IL-1 β released in group B were comparable on day 1 [805.084 ± 142.97414 pg/mL] and day 4 [828.186 ± 175.7136 pg/mL], with reductions in IL-1β levels observed at day 10 [53.360 ± 27.6245 pg/mL] (ie, after initiation of JNJ-54175446 on day 4) (Supplemental figure 5)
